# Pre-eruptive magmatic processes re-timed using a non-isothermal approach to magma chamber dynamics

**DOI:** 10.1038/ncomms12946

**Published:** 2016-10-05

**Authors:** Chiara Maria Petrone, Giuseppe Bugatti, Eleonora Braschi, Simone Tommasini

**Affiliations:** 1 Department of Earth Sciences, The Natural History Museum, Cromwell Road, SW7 5BD London, UK; 2 Instituto di Geoscienze e Georisorse, CNR, Sezione di Firenze, Via La Pira 4, 50121 Firenze, Italy; 3 Dipartimento di Scienze della Terra, Università degli Studi di Firenze, Via La Pira 4, 50121 Firenze, Italy

## Abstract

Constraining the timescales of pre-eruptive magmatic processes in active volcanic systems is paramount to understand magma chamber dynamics and the triggers for volcanic eruptions. Temporal information of magmatic processes is locked within the chemical zoning profiles of crystals but can be accessed by means of elemental diffusion chronometry. Mineral compositional zoning testifies to the occurrence of substantial temperature differences within magma chambers, which often bias the estimated timescales in the case of multi-stage zoned minerals. Here we propose a new Non-Isothermal Diffusion Incremental Step model to take into account the non-isothermal nature of pre-eruptive processes, deconstructing the main core-rim diffusion profiles of multi-zoned crystals into different isothermal steps. The Non-Isothermal Diffusion Incremental Step model represents a significant improvement in the reconstruction of crystal lifetime histories. Unravelling stepwise timescales at contrasting temperatures provides a novel approach to constraining pre-eruptive magmatic processes and greatly increases our understanding of magma chamber dynamics.

## Abstract (web summary)

Zoned crystals may reflect magma chamber dynamics, where changes in temperature, pressure and timescale before volcanic eruption may be measured. Petrone *et al*. develop a new Non-Isothermal Diffusion Incremental Step model to reconstruct crystal lifetime histories to constrain pre-eruptive magmatic processes.

## Graphical abstract

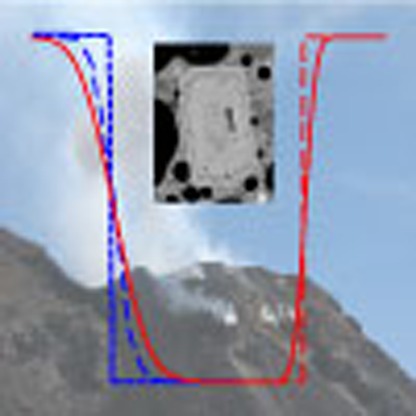

It has long been known that the timescale of magma formation, storage and ascent beneath active volcanoes is the key to constrain pre-eruptive magmatic processes and magma chamber dynamics, which provide the basis for volcanic hazard assessment (for example refs 1, 2, 3, 4, 5, 6, 7). Temporal information is locked in the chemical zoning profile of crystals in erupted volcanic products. Stepped compositional zoning in such crystals is the result of physico-chemical variations in the magma (for example refs 8, 9) arising from several processes including cooling, degassing, magma mixing and magma assimilation^9^,^10^,^11^. The most dramatic effect on mineral texture and composition is caused by magma mixing, which results in reverse, oscillatory and patchy zoning^9^,^10^,^12^. The degree of interaction between magmas strongly depends on the relative contrast in their viscosity, temperature, composition and volume^13^,^14^,^15^,^16^. Elemental diffusion within initially zoned minerals can smooth and even obliterate initial zoning profiles depending on the time that the crystal spent at high temperature and the elemental diffusion rate. Magma eruption freezes the smoothing of the stepped compositional zoning profile, thus allowing temporal information to be unlocked by means of diffusion chronometry (for example refs 17, 18, 19, 20, 21, 22, 23).

Diffusion coefficients (*D*) in silicates are controlled by a number of parameters such as temperature, pressure, water and oxygen fugacity, chemical composition and crystallographic direction. As it has already been shown (for example refs 20, 23, 24), temperature is by far the most critical parameter because diffusion is a thermally activated process. Thus, the variation of the diffusion coefficient strongly depends on the magnitude of the activation energy of the element in a given crystal and the temperature^20^. Clearly, it is critical to accurately assess the temperature at which diffusion took place in order to place meaningful constraints on the timescale of pre-eruptive processes. In case of magma mixing, the temperature contrast can be as high as 300 °C (ref. 25). If there is more than one event of magma mixing during crystal growth, or crystals have multiple contacts with magmas at different temperatures (that is, multi-stage process), the rate of elemental diffusion within crystals can be significantly affected^26^, resulting in severe consequences for the calculated residence time of the crystals. This could be the case of multi-zoned clinopyroxenes with high-Mg# (Mg/(Mg/Fe) at.) compositional bands, formed in the internal portion of the crystal as a result of magma chamber replenishment by hotter and more mafic magma, intercalated with low Mg# core and rim portions formed in equilibrium with the resident magma at lower temperature^27^,^28^,^29^,^30^,^31^. In these crystals, elements will thus diffuse at a higher rate across the core-band boundary than across the band-rim boundary.

Kinetic modelling of compositional zoning in olivine crystals has been used to yield information on crystal (and magma) residence time in different portions of magmatic plumbing systems characterized by different conditions or magmatic environments^21^,^22^. Each compositional boundary experienced isothermal diffusion on contrasting timescales in different magmatic reservoirs, with the temperature set at the pre-eruptive equilibrium condition, in agreement with the observed core-rim zoning patterns of the studied olivine crystals^21^,^22^. A two-step diffusion model has been recently used to constrain residence time of single portions of plagioclase crystals in equilibrium with liquids having different compositions and temperatures in the plumbing system of the Santorini volcano (Greece)^6^. In addition, a combination of U-series data, crystal size distribution and trace element zoning has been recently used to link the storage time to the thermal state of a magma body^32^. However, the direct link between crystal residence time at different temperatures in active volcanoes (that is, the higher the temperature, the higher the diffusion rate^26^) has not been fully explored. Indeed, when more than one compositional boundary is present (that is, multi-stage evolution), it is important to take into account that elemental diffusion rates of the internal and external compositional boundary layers can be significantly different, depending on temperature contrast and elemental activation energy for a given crystal. This is critical to diffusion chronometry studies aiming to establish meaningful crystal residence times and set constraints on magma chamber dynamics.

Here we propose a novel diffusion chronometry approach that explicitly accounts for the non-isothermal nature of complex crystal compositional zoning in volcanic systems. Our new Non-Isothermal Diffusion Incremental Step (NIDIS) model considers different diffusion coefficients for each crystal compositional boundary to match its specific equilibrium temperature. This permits reconstruction of the lifetime history of complex compositionally zoned crystals, and contributes to a better understanding of magmatic storage and pre-eruptive processes in the plumbing systems of active volcanoes.

## Results

### The non-isothermal conceptual approach of the NIDIS model

**Figure 1:**
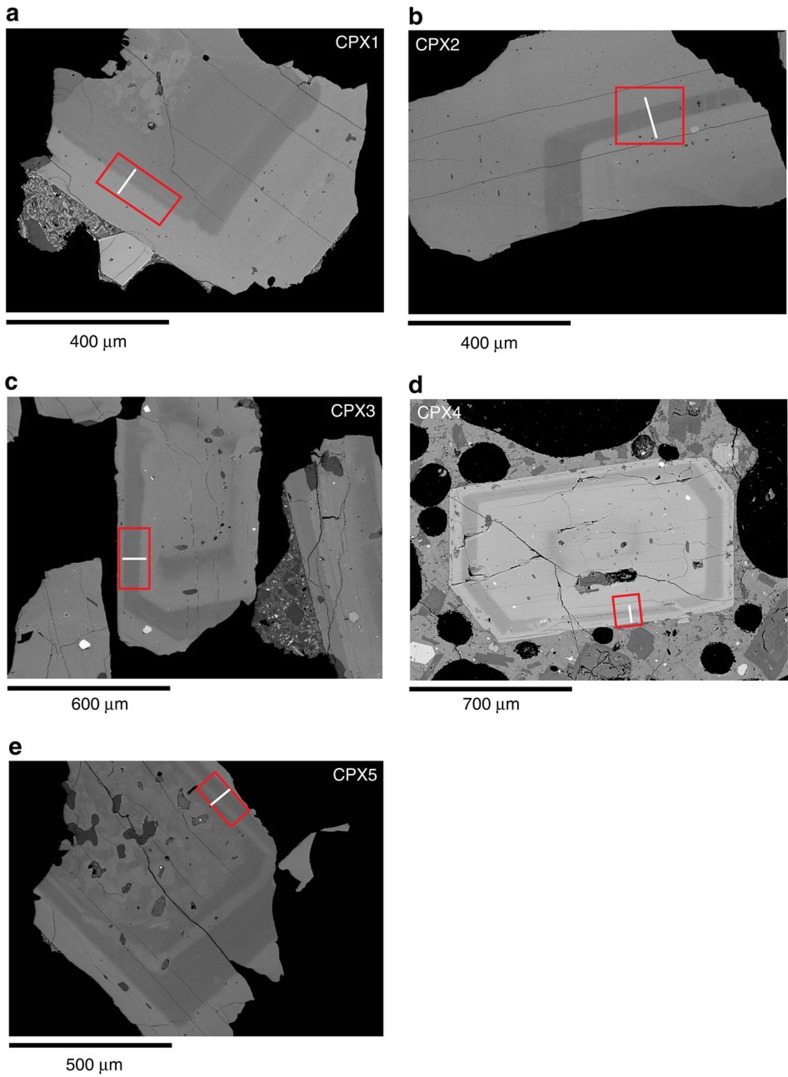
Zoned clinopyroxene. High-resolution back-scattered electron (BSE) images of selected clinopyroxene crystals of Stromboli volcano modelled in this study. The red boxes indicate the area where the grey scale raster has been determined using the ‘greyscale calculation’ described in Methods, whereas the white line is where the electron microprobe profile has been measured (Methods). (**a**) clinopyroxene 1 (CPX1); (**b**) clinopyroxene 2 (CPX2); (**c**) clinopyroxene 3 (CPX3); (**d**) clinopyroxene 4 (CPX4); (**e**) clinopyroxene 5 (CPX5).

**Figure 2:**
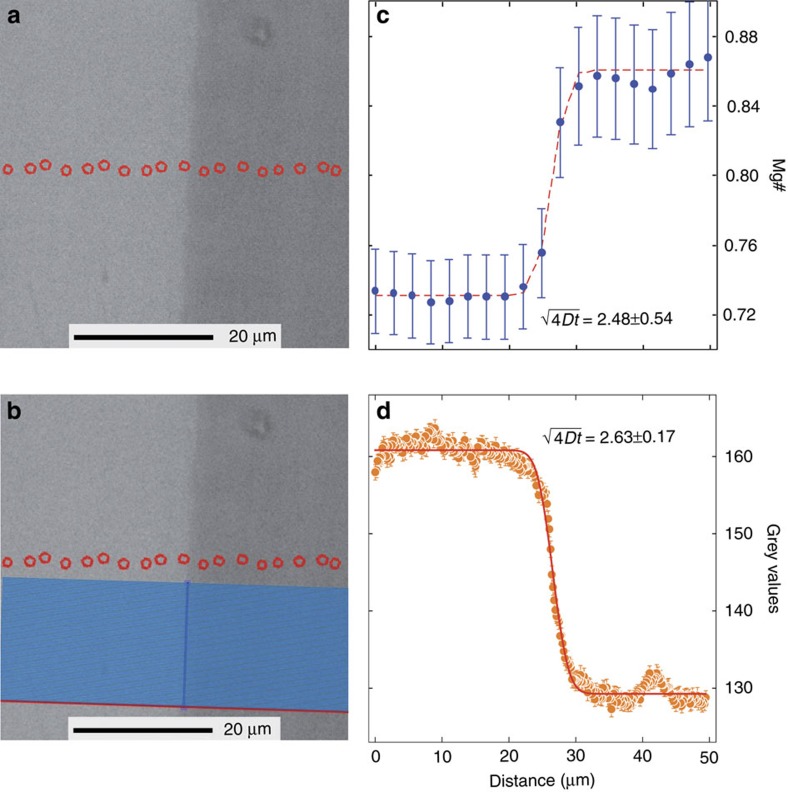
Electron microprobe versus greyscales profile. The electron microprobe compositional profile (**a**,**c**) is used to calibrate the grey scale profile (**b**,**d**) along the clinopyroxene compositional boundary. We used BSE greyscale values as a proxy of Mg# variations in clinopyroxene on the basis of the identical, within error, fitting parameter (that is, 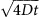
=2.63±0.17 for the grey value and 2.48±0.54 for Mg#) obtained using both the greyscale (red line in **d**) and Mg# (red line in **c**) diffusion profile (Methods). (**a**) BSE-SEM image of the compositional boundary of a clinopyroxene analysed in this study. The red dots mark the single point analysis of the electron microprobe profile; (**b**) Same image as in **a** showing the grey values raster (blue lines) calculated parallel to the electron microprobe profile (red dots), using the ‘greyscale calculation’ code (Methods). The raster (light parallel blue lines, the first line of the raster is reported as single red line) is perpendicular to the guideline (single bold blue line) drawn parallel to the boundary layer (Methods); (**c**) Mg# versus distance (μm) compositional profile, along the traverse (red dots in **a**), of the clinopyroxene shown in **a**. The red line is the best fitting of the compositional profile. Error bars (2 s.d.) in Mg# values represent error propagation uncertainties of electron microprobe analyses; (**d**) Grey value versus distance (μm) compositional profile, along the raster (blue lines in **b**) of the clinopyroxene shown in **b**. The red line is the best fitting of the diffusion profile as calculated with the NIDIS. The grey value profile is the average of 100 profile lines; error bars represent the 2 standard error of the mean (2 s.e.).

The model has been developed on the basis of diffusion profiles derived from high-resolution back-scattered electron (BSE) images of clinopyroxenes (Fig. 1), rather than electron microprobe analyses. To constrain a meaningful diffusion chronometry, each BSE image has been calibrated with the actual compositional zoning of clinopyroxenes obtained via electron microprobe (Fig. 2; Methods and Supplementary Figs 1–6). The correspondence between the grey value profile from BSE image (that is, brightness) and the Mg# profile (Fig. 2) was tested by calculating the fitting parameter 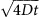 (see Methods) to the diffusion profile of Mg# (Fig. 2c) and grey values (Fig. 2d). The result is identical within error, and demonstrates that the grey value profile from BSE image can be used as a proxy of Mg# variation in clinopyroxene, provided that Ca variation across the zoned boundary is negligible compared to Fe and Mg. This has been previously shown by Morgan *et al*.^17^ for clinopyroxene from Vesuvius, and is further explored here (Methods and Supplementary Fig. 1). Therefore, in similar future studies, only a few electron microprobe spot analyses concentrated on the plateaux of each crystal’s compositional portion will be necessary, significantly reducing analytical time. In the example reported in Fig. 2, the lighter (∼160) and the darker (∼128) grey values correspond to Mg# ∼0.73 and ∼0.86 respectively. The compositional zoning profile is the result of diffusion, and not of growth, as illustrated by the profile of a slowly diffusing element such as Ca, which provides evidence of having maintained the initial boundary condition, that is no diffusion occurred for Ca (Supplementary Fig. 1).

Key advantages of working with greyscale values diffusion profiles include: improved spatial resolution (<0.5 μm versus >2–3 μm) (Fig. 2); the ability to measure hundreds of profiles across the zoned boundary, thus resulting in an average profile with low uncertainty (that is, 2 standard error of the mean, Fig. 2d); rapid data acquisition compared with microprobe compositional profiles (<10 min acquisition time for a high-resolution of 2,000 dpi BSE image versus ∼2 h for 30 points in a microprobe profile); and a significant reduction of the convolution effect of microprobe analyses^33^. The increased spatial resolution yields a significant improvement on the error of the fitting parameter 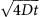 (Fig. 2c,d), in particular when the length of the diffusion profile is only a few microns (for example, some 5 μm, Fig. 2).

**Figure 3:**
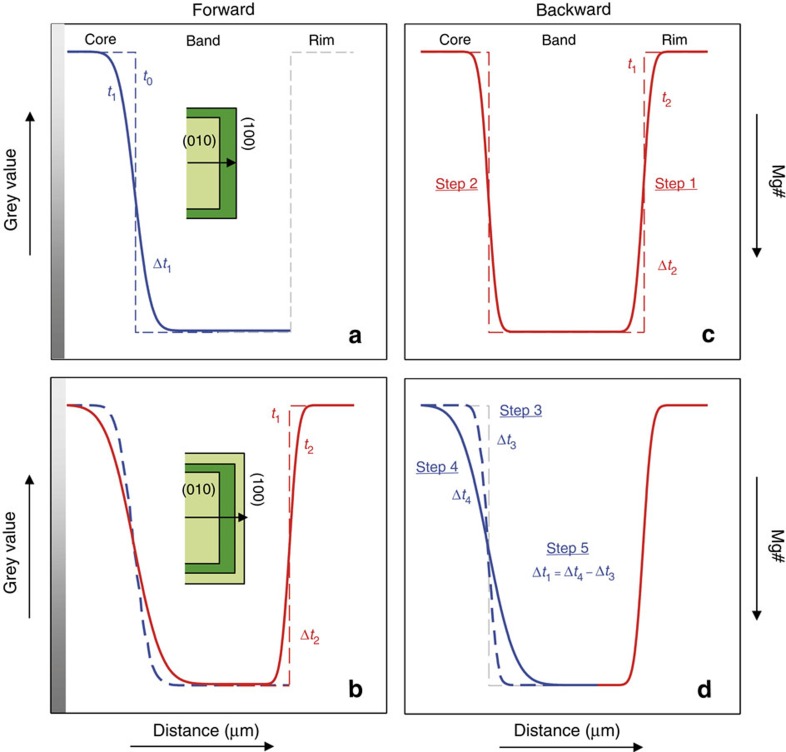
Conceptual description of the Non-Isothermal Diffusion Incremental Step model. Fe–Mg compositional zoning profiles are measured perpendicular to the (100) plane (black arrow in **a**,**b**) of the schematic clinopyroxene (light green=low Mg#—high grey value; dark green=high Mg#—low grey value). *Y*-axis=contrast in grey value and Mg# (left and right hand side) across the boundary layers; *x*-axis=extent of diffusion in μm. Forward model: (**a**) development of the compositional zoning from the instantaneous dark band growth at *t*_0_ (dashed blue line) to the time *t*_1_ spent at *T1* °C (solid blue line). The dashed grey line represents the initial dark band—rim conditions yet to be formed; (**b**) clinopyroxene rim instantaneous growth at *t*_1_ (dashed red line) and proceeding Fe–Mg diffusion at *T*2 °C (solid red line), with *T*2<*T*1, until the eruption at *t*_2_. Fe–Mg diffusion is not limited to the dark band—rim boundary, but also affects the core—dark band boundary. The dashed blue line corresponds to the diffusion profile developed at *T1* °C in the *Δt*_1_ time span (solid blue line in **a**). Backward model: (**c**) the dashed red line represents the initial boundary conditions of Step 1 and 2 at *T*2 °C, whilst the solid red line is the core—dark band—rim Fe–Mg diffusion profile at *T*2 °C developed during *Δt*_2_; (**d**) the solid red line is the final dark band—rim profile developed during Step 1 and corresponds to the compositional profile in **b**, whilst the solid blue line is the final Fe–Mg diffusion profile developed at *T*1 °C during *Δt*_4_, and corresponds to the actual compositional profile in **b**. The initial core—dark band conditions are represented by the dashed grey line. The dashed blue line is the fictitious Fe–Mg diffusion profile developed at *T1*°C (>*T*2), during *Δt*_3_ and corresponds to the profile developed at *T*2 °C in **c** (Step 2, solid red line). The clinopyroxene resident time at *T*1 °C is *Δt*_1_=*Δt*_4_−*Δt*_3_, and the total crystal residence time is given by *Δt*_1_+*Δt*_2_.

The clinopyroxene crystals in Fig. 1 have multiple compositional bands, as testified by different greyscale values of BSE images, which are correlated to Mg# variation (Fig. 2, Methods and Supplementary Figs 2–6). The darker grey values (high Mg#) record mineral growth in a hotter and more mafic magma, whereas the lighter grey values (low Mg#) record mineral growth in a cooler and more evolved magma, resulting in a final diffusion profile at different temperatures as conceptually described in Fig. 3. The rationale of the NIDIS model is to deconstruct the entire core-rim diffusion profile into distinct isothermal steps with their own diffusion coefficients, each step taking into account the diffusion timescale of the previous step. This approach is necessary for complex crystals with more than one compositional boundary layer and thus showing a multi-stage evolution (for example, CPX2, CPX3, CPX4, CPX5, Fig. 1). However, it can be also used for two-stage evolution cases where there is only one compositional boundary (for example, CPX1, Fig. 1).

The crystallization temperature of each clinopyroxene portion in Fig. 1 is based on extensive research on Stromboli volcano (for example refs 28, 29, 30, 31), along with a cross-check using a clinopyroxene-liquid geothermometer^34^ (Methods) to test the absolute temperature value and the equilibrium with the liquid. The estimated temperatures (1098±15 °C and 1150±10 °C, see below) permitted the determination of different Fe–Mg diffusion coefficients across the boundary using the thermodynamic data of Dimanov and Sautter^35^ (Methods). Considering that clinopyroxene growth rate is some orders of magnitude faster than Fe–Mg diffusion rate (for example refs 35, 36), we can delineate the following lifetime history of clinopyroxene crystals from nucleation to final quenching upon eruption (Fig. 3a,b). At time *t*_0_ the high Mg# band around the low Mg# core grows almost instantaneously (dashed blue line, Fig. 3a). During the time interval *Δt*_1_=*t*_1_–*t*_0_ Fe–Mg diffusion across the boundary takes place, with a diffusion coefficient *D*1 determined by the temperature *T*1 of the magma in which the high Mg# band formed (solid blue line, Fig. 3a). At time *t*_1_ the crystal is transferred to another portion of the magma chamber characterized by a temperature *T*2<*T*1, and the low Mg# crystal rim grows (dashed red line, Fig. 3b). The time interval of this diffusion step is given by *Δt*_2_*=t*_2_*−t*_1_, with *t*_2_ corresponding to magma eruption. During this time interval, Fe–Mg exchange proceeds across the entire crystal (that is, from low Mg# core to the high Mg# band and the low Mg# rim) with a Fe–Mg diffusion coefficient *D*2<*D*1, because of the lower temperature, and determines the final diffusion profile (solid red line, Fig. 3b) of the crystal lifetime history.

To estimate the timescale (*Δt*_1_+*Δt*_2_) producing such a profile, we can use the analytical solution of diffusion developed for a semi-infinite plane sheet (equation 2.14; ref. 37), which is based, however, only on the initial boundary conditions (dashed blue and red lines, Fig. 3a,b) and a single diffusion coefficient. In contrast, the crystal lifetime history delineated in Fig. 3a,b clearly implies two different diffusion coefficients for the core-band boundary layer (*D*1 at *T*1 and *D*2 at *T*2).

To overcome this problem we used a backward approach and estimated the timescale *Δt*_1_ (Fig. 3a) by difference. The following backward method (Fig. 3c,d) has been developed always starting, in each step, from the initial boundary conditions (dashed blue and red lines, Fig. 3a,b) implicit in the analytical diffusion equation across a semi-infinite plane sheet^37^. In Step 1 (Fig. 3c), the timescale *Δt*_2_ to reproduce the diffusion profile across the high Mg# band and the low Mg# rim is estimated using the diffusion coefficient *D*2 (lifetime history *Δt*_2_, Fig. 3b). In Step 2 (solid red line, Fig. 3c) we determined the diffusion profile developed during *Δt*_2_ across the low Mg# core and the high Mg# band, using the diffusion coefficient *D*2. In Step 3 (dashed blue line, Fig. 3d) a fictitious timescale *Δt*_3_ is estimated in order to reproduce the diffusion profile developed during Step 2 across the low Mg# core and the high Mg# band (solid red line, Fig. 3c), using the diffusion coefficient *D*1, that is assuming that it formed at temperature *T*1. In Step 4 (solid blue line, Fig. 3d) we calculated another fictitious timescale *Δt*_4_ to reproduce the diffusion profile across the low Mg# core and the high Mg# band, using the diffusion coefficient *D*1, that is assuming that it formed entirely at temperature *T*1. Eventually, we could obtain the timescale *Δt*_1_ by difference (Step 5, *Δt*_4_−*Δt*_3_) reflecting the lifetime of the clinopyroxene crystal in the magma body at *T*1 (Fig. 3a). This diffusion chronometry procedure yields the total crystal residence time within the magma reservoir summing up *Δt*_1_ (time spent at *T*1, Step 5 of Fig. 3d) and *Δt*_2_ (time spent at *T*2, Step 1 of Fig. 3c).

### NIDIS and the lifetime history of Stromboli clinopyroxene

We applied the NIDIS model to carefully screened (Methods) compositionally zoned clinopyroxene of Stromboli volcano (Italy) from the present-day activity (<2000 years). The stratovolcano of Stromboli (ca. 300 km^3^) is one of the most famous and best studied volcanoes worldwide because of its continuous and moderately explosive ‘Strombolian’ activity over, at least, the last 2000 years (for example refs 38, 39). The present day activity is moderately explosive and persistently erupts bombs, black scoriae, lapilli and ash (ca. 4–5 events per hour). Lava flows are periodically erupted (some 15 episodes in the last 100 years). The typical ‘Strombolian’ activity consists of High Porphyritic (HP) lavas and black scoriae, formed of phenocrysts of olivine (4–8 vol%), clinopyroxene (12–20 vol%) and plagioclase (20–25 vol%). This activity is occasionally interposed with more violent eruptions and the ejecta consist of HP scoriae intermingled with highly vesiculated yellowish Low Porphyritic (LP) scoriae (the so-called golden pumice). The LP scoriae have a low phenocryst content (ca. 5 vol%) and consist of microphenocrysts of olivine, clinopyroxene and rare plagioclase.

There is compelling geochemical and radiogenic isotope evidence that the LP scoria represents the fresh, phenocryst-poor magma feeding the volcanic system of Stromboli that upon storage, crystallization and homogenization within the magma chamber, forms the magma erupted as HP lava and scoriae. The relatively constant composition of the HP magma demonstrates negligible crystal removal during crystallization along with efficient mixing (that is, homogenization time<residence time), forming a steady-state, compositionally homogeneous reservoir. Studies of Stromboli^28^,^29^,^30^,^31^ have clearly established the intensive parameters of both the LP and HP magmas. The LP magma has *T*=1150±10 °C, *P*=100–270 MPa, H_2_O=3.0±1 wt%, *f*O_2_=NNO+0.5 (NNO, Nickel-Nickel Oxide buffer), whereas the HP magma has *T*=1098±15 °C, *P*<100 MPa, H_2_O=0.2 wt%, *f*O_2_=NNO+1.

**Figure 4:**
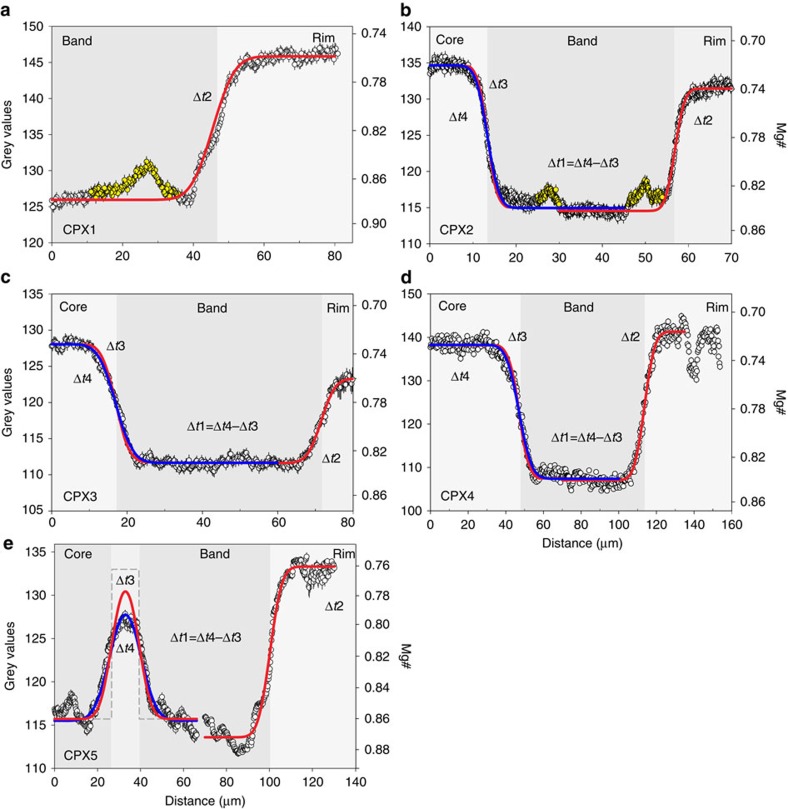
Results of Non-Isothermal Diffusion Incremental Step model. Grey value diffusion profiles (open circles) of the 5 analysed clinopyroxenes (**a**, CPX1; **b**, CPX2; **c**, CPX3; **d**, CPX4; **e**, CPX5) along the red boxes of Fig. 1. Mg# is determined by microprobe analysis (Methods, Supplementary Figs 2–6) and reported for reference on the basis of Fig. 2 (Methods). The dark band testifying the arrival of the new mafic magma is shaded in dark grey. Each diffusion profile is the average of 200–600 lines across the boundary layers and is calculated using the ‘greyscale calculation’ procedure (Methods). The error bar of each point represents the 2 standard error of the mean. The yellow circles are points that have not been used for the fitting procedure due to the occurrence of intrinsic banding (see text). Fe–Mg diffusion profiles in CPX1–4 across the two boundaries (one boundary for CPX1) have been modelled using the analytical equation developed for a semi-infinite plane sheet (equation 1), whereas Fe–Mg diffusion profile in CPX5 had been modelled using the analytical solution developed for diffusion within a finite reservoir of limited extend (equation 2). Solid lines are the result of the NIDIS model and represent the best fitting grey value diffusion profiles of the core—dark band (blue line at 1150 °C) and dark band—rim (red line at 1098 °C). The solid red lines on the core-dark band represent the diffusion profile developed during *Δt*_2_ (see also Fig. 3c) and correspond to the fictitious profile developed during *Δt*_3_ at 1150 °C (see also Fig. 3d). Grey dashed line in CPX5 (**e**) represents the inferred initial boundary condition of the finite reservoir of limited extend (equation 2), whilst the solid red and blue lines of the finite reservoir represent, as for other clinopyroxenes, the profiles at *Δt*_3_ and *Δt*_4_ respectively (see text for details). The different *Δt*_1–4_ are appropriately referred to into the text.

On the basis of BSE images, all the selected clinopyroxenes have compositional bands (Fig. 1) reflecting crystallization from magmas with different Mg#. Clinopyroxene 5 (Fig. 1, CPX 5) records the most complex lifetime history with a resorbed low Mg# core (light grey) surrounded by a high Mg# portion (dark grey) and rimmed by a low Mg# band, another high Mg# band and eventually a low Mg# rim. All the light and dark grey clinopyroxene portions have similar low and high Mg# (0.72–0.76; 0.83–0.88), respectively (Fig. 4), showing the occurrence of crystal exchange between the two domains of the magma chamber resulting from, for example, convective overturning and stirring (for example refs 16, 40, 41, 42, 43) during homogenization of the LP feeding magma within the HP resident magma.

We have applied the Putirka^34^ geothermometer to the clinopyroxenes in Fig. 1 and verified that the high-Mg# band and the low-Mg# core and rim are in equilibrium with the LP and HP magma, respectively, yielding temperature estimates consistent with those reported by the abovementioned studies achieved by other techniques (for example, melt inclusions, experimental petrology).

Temperatures of 1150 °C (LP magma) and 1098 °C (HP magma) yield different Fe–Mg diffusion rates^35^ between the core—dark band (Fig. 3a,d) and the dark band—rim (Fig. 3b,c). Thus, the modelled greyscale profiles (Fig. 4) result from different Fe–Mg diffusion steps at 1150 °C (blue lines) and 1098 °C (red lines).

Fe–Mg diffusion profiles in clinopyroxenes 1–4 (Fig. 1, CPX1–4) across the two boundary layers have been modelled (Fig. 4, CPX1–4) using the analytical equation developed for a semi-infinite plane sheet (equation 2.14, ref. 37; equation 1). The Fe–Mg diffusion profile in clinopyroxene 5 presents the most complex situation with a resorbed low Mg# core, surrounded by a high Mg# portion, and rimmed by a low Mg# band, a high Mg# band and a low Mg# rim (Fig. 1, CPX5). In particular, the low Mg# band situated between the two high Mg# bands lacks any compositional plateau, and is characterized by a bell shape (Fig. 4, CPX5). This case cannot be treated as a semi-infinite reservoir but requires the analytical solution developed for diffusion within a finite reservoir of limited extent (equation 2.15, ref. 37; equation 2). Determining the initial boundary condition (that is, the initial shape of the compositional profile^20^,^44^,^45^) is not straightforward. Common strategies include using an arbitrary maximum concentration range and comparing zoning profile of elements with different diffusivities^20^,^44^,^45^. For clinopyroxenes 1 through 4, the maximum and minimum grey values (or Mg#) correspond to the plateau exhibited by the diffusion profiles (Fig. 4, CPX1–4). In the case of clinopyroxene 5, there is no plateau in the low Mg# portion between the two high Mg# bands (Fig. 4, CPX 5), although we can safely assume that the bell-shaped low Mg# portion had the same Mg# (or grey value) of the rim given the occurrence of only two magmatic domains at Stromboli (LP and HP magmas).

The analytical solution (equations 1 and 2) of the modelled diffusion profiles are reported in Table 1 in terms of error function fitting parameters at 95% confidence levels, nonlinear least squares (*r*^2^), timescales for each diffusion step and the total pre-eruptive crystal residence time. Two different time estimate uncertainties are given: 2 s.d. is the calculated error propagation considering the uncertainties on both temperature (±10 °C and ±15 °C) and the fitted 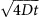 parameter (*D* diffusion coefficient, *t* time elapsed, equations 1 and 2); 2 s.d.* is the calculated error propagation considering only the uncertainty on the fitted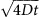 parameter. In all modelled profiles, there is a robust and excellent fitting with *r*^2^ between 0.9783 and 0.9962 (Table 1).

The diffusion chronometry derived by the modelled compositional profiles using greyscale values of BSE images as a proxy of Mg# variation (Fig. 4), provides evidence for a residence time of a few years (*Δt*_2_) at the temperature of the HP magma (1098 °C) and an extremely short residence time (*Δt*_1_) at the temperature of the feeding LP magma (1150 °C) for all the studied clinopyroxenes (Fig. 4, Table 1). Considering the propagated error on *Δt*_1_, the residence time at high *T* is almost instantaneous (Table 1). The total crystal residence time before eruption varies from 1.8 (CPX2) to 11.9 years (CPX1) after the growth of the low Mg# rim at lower *T* (Fig. 4, Table 1). Only one compositional boundary (band-rim) could be modelled in clinopyroxene 1 (low *T* diffusion, Fig. 4a), implying that its residence time should be considered a minimum value.

The calculated total residence time for clinopyroxene 5, the diffusion profile of which has been modelled using the analytical solution for a finite reservoir of limited extent (equation 2), is 11.8 years (Table 1), similar to the other clinopyroxenes, and the deconstruction of the lifetime history indicates a relatively short time, albeit slightly longer than the other cases, spent at the temperature of the feeding LP magma (Table 1). As in the case of clinopyroxene 1, the total residence time should be considered a minimum estimate, since the time elapsed from inner core entrapment and resorption within the high-T magma cannot be determined.

### Uncertainties and errors in the NIDIS approach

Uncertainties and source of errors in diffusion chronometry modelling have been explored at length in the literature and an excellent review can be found in Costa *et al*.^20^. Uncertainties, errors and pitfalls are connected with crystal orientation and anisotropy of diffusion, initial conditions, diffusion coefficients and temperature.

It has long been known that diffusion in anisotropic crystals is a vector property and that using a diffusion coefficient inappropriate for the direction of the measured diffusion profile will yield incorrect timescales^20^. We measured all our diffusion profiles along the direction perpendicular to the (100) crystal plane of selected crystals cut parallel to (010) (Figs 1, 3 and 4) and we used the corresponding clinopyroxene diffusion coefficients^35^ (Methods). The initial boundary conditions have been determined on the basis of compositional plateaux (Figs 2 and 4) and are supported by the chemical profiles of slow diffusing elements such as Ca (Methods and Supplementary Fig. 1), providing a robust indication that the observed profiles are the result of diffusion and not kinetic/growth processes.

The careful screening of the choice of proper clinopyroxene cuts has certainly mitigated the source of uncertainties related to the crystal habit parameter. However, the principal factor affecting error propagation on timescale in all diffusion chronometry studies, and our NIDIS model is not an exception, is the uncertainty on the temperature (Eq. 4). As pointed out by Costa *et al*.^20^, the uncertainty on temperature is reflected in an uncertainty on diffusion coefficient estimates, and hence timescales, as a function of the activation energy and the specific temperature. Errors increase with increasing activation energy and decreasing temperatures^20^. The higher activation energy of Fe–Mg in clinopyroxene than in other crystals determines a greater variation of the diffusion coefficient (*D*) with *T*: the difference in *D* between 1150 °C and 1098 °C is ∼74% with an activation energy of 406 kJ mol^−1^ (ref. 35), while it is ‘only’ 48% with a nominal activation energy of 200 kJ mol^−1^ (for example, olivine, plagioclase). The high sensitivity of clinopyroxene Fe–Mg exchange with temperature makes it more apt than other crystals to detect timescale estimates in response to small *T* variations (for example, 50 °C in our case) in volcanic systems.

We considered an error of ±10 °C for the high-T LP magma and ±15 °C for the low-T HP magma, respectively (2 s.d.; refs 28, 29, 30, 31), which is reflected in the propagated error on *D* estimates of some 24 and 39%, respectively (Table 1). This translates into a maximum of some 40% uncertainty in the total residence time estimate (for example 11.9±4.8 years, CPX1, Table 1) when coupled with the uncertainty in the 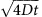 parameter (2 s.d., Table 1). The uncertainty is reduced by a factor > 2 if only the uncertainty in the 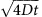 parameter (2 s.d.*, Table 1) is considered. Our NIDIS method reduces the error on diffusion chronometry modelling by using the most accurate mathematical fitting, and by minimising the error related to crystal orientation and greyscale calculation (Methods), although temperature estimation still remains the largest source of uncertainty. We are confident that advances in the knowledge of the thermodynamic properties of silicate minerals will lead to the development of more accurate geothermometers and help to minimize this single major source of error in future.

## Discussion

The total crystal residence time (Table 1) for clinopyroxene diffusion profiles of the present-day activity at Stromboli (Fig. 4) provides timescale information on the magma plumbing system. The total clinopyroxene residence time of a few years (*Δt*_1_+*Δt*_2_, Table 1) in the Stromboli magmatic system is consistent with timescale estimates obtained by radiogenic isotope approaches (Sr and U-series isotopes^27^,^46^). However, the most striking result of our novel approach is the almost instantaneous residence time (*Δt*_1_, Table 1) at high *T* (ca. 1150 °C) of the analysed clinopyroxenes in comparison to the residence time within the low-T HP reservoir (*Δt*_2_, Table 1). This result is a unique output of the NIDIS model, which could not be deciphered using radiogenic isotope approaches. This timescale sets robust constraints on magma chamber dynamics indicating a well mixed and stirred reservoir where the inputs of new and fresh LP magma are rapidly homogenized within the resident HP magma^27^ at lower T (ca. 1098 °C). The second order, low amplitude, grey value (or Mg#) intrinsic banding^47^ observed on the dark band plateau of clinopyroxenes 1 and 2 (Fig. 4, CPX1 and CPX2 yellow circles) are likely related to kinetic processes^48^. When crystal growth is too rapid, elemental diffusion is not able to maintain equilibrium at the solid–liquid interface. Consequently, Mg is depleted more than Fe during clinopyroxene crystallization in the liquid around the growing crystal, causing the successive growth of the relatively lower Mg# (or higher grey value) intrinsic banding. Given that the temperature difference between the input of the fresh LP magma (ca. 1150 °C) and the resident HP magma (ca. 1098 °C) is probably constant, the reason why the profiles of clinopyroxenes 3 and 4 do not have any intrinsic banding could be related to the mass fraction of the input magma with respect to the volume of the magmatic reservoir (<0.3 km^3^, ref. 27). The lower the mass fraction of input magma, the higher the undercooling and the clinopyroxene growth rate^36^, leading to the development of intrinsic banding. The occurrence of intrinsic banding could thus be an indirect record of the magma supply rate in well stirred and homogeneous magma chambers of active volcanoes.

Finally, the diffusion profile of clinopyroxene 5 (Fig. 1, Fig. 4, CPX5) presents the most complex lifetime history of crystal transfer between the LP and HP magmas. The inner core formed in the HP magma reservoir, was successively entrapped and resorbed in the LP feeding magma, and the resorbed core acted as seed to form the first ∼25 μm high Mg# portion. The crystal was then drawn back in the HP magma, growing ∼12 μm of the low Mg# portion (that is the finite reservoir) and was then entrapped again in the LP magma forming the ∼60 μm high Mg# portion. The timescale of crystal growth of these last two events was almost instantaneous given that the diffusion profile of the low Mg# portion is symmetric on both sides. The crystal spent a couple of years (*Δt*_1_, Table 1) in this reservoir, allowing Fe–Mg diffusion to operate. Eventually the crystal was transferred to the HP magma where it grew the last ∼60 μm rim over a timescale of some 9 years (*Δt*_2_, Table 1) until eruption.

The NIDIS model has been illustrated using the zoned clinopyroxenes of Stromboli volcano (Fig. 4) and yields a novel approach to constrain the timescale of the stepwise history of crystals in the realistic scenario of diffusion chronometry at two different temperatures. The rationale of the NIDIS model (for example, diffusion chronometry at different temperatures) and the developed Matlab code (Methods) can be applied to virtually any volcanic system and mineral phase. For example, the NIDIS model can resolve BSE diffusion chronometry profiles such as Fe–Mg in orthopyroxene and olivine and CaAl–NaSi in plagioclase, and Ti in quartz using cathodoluminescence^49^. Caution needs to be exercised on a case by case basis considering: the rate of diffusion of the elements involved (that is, the timescale range that can be detected); the contribution of each element to the yield of BSE images (for example, negligible Ca variation allowing use of the grey value as a proxy for Mg# in the case of clinopyroxene, Methods); other limiting factors such as meeting the criteria for choosing diffusion profiles^20^,^44^,^45^; availability of diffusion coefficients; diffusion profiles solvable with BSE images; and appropriate geothermometers or software packages for temperature estimation (for example refs 50, 51). Provided these considerations are satisfied, the advantages of working with BSE images are manifold with respect to electron microprobe data.

In conclusion, the NIDIS model represents a major improvement in crystal residence time estimates, and the deconstruction of the lifetime history of crystals in non-isothermal, time-constrained steps provides a new approach to resolve pre-eruptive magmatic processes. The implications of the NIDIS model in terms of resolving the relative difference in crystal residence time between high-T and low-T magmatic environments are far-reaching for our understanding of magma chamber dynamics.

## Methods

### Greyscale calculation

The greyscale values of the compositional zoned interface for each crystal were calculated using a high-resolution back-scattered (BSE) SEM image and our new Matlab script greyvalues. BSE images were acquired with the LEO 1445 VP SEM, at the Imaging and Analysis Centre of The Natural History Museum (IAC-NHM), operating at 15 KeV and 100 μA electron current, 460 nm spot size and 14 mm WD (working distance). The images were acquired with the INCA software accumulating eight frames of 2046 × 1536 pixels each. To obtain the best results from diffusion modelling in terms of reducing the orientation effect of the crystal and minimize the effect of not sectioning perpendicular to the core-rim boundary, careful screening was carried out in the analysed thin sections. Over 100 clinopyroxene crystals have been examined by petrographic microscope and we selected only those crystals cut parallel to the (010) crystallographic plane based on the crystal shape (that is, Fig. 1), cleavage and interference colours. The diffusion profiles have been acquired along the direction perpendicular to the (100) crystallographic plane (Figs 2 and 3).

The Matlab greyvalues script allows extrapolation and export of grey values from a BSE image. Being designed for straight features, the program asks the user to draw a guideline (Fig. 2b) along the straight feature of interest (for example, boundary between two zones of a crystal). It then allows interactive selection of the length of the profile lines (perpendicular to the guideline, Fig. 2b) along which the grey values will be extrapolated. Once the profile line is set, the program extrapolates the grey values along as many profile lines as the length in pixels of the guideline (we calculated a minimum of 200 lines up to >600). The grey values are calculated using the nearest neighbour colour interpolation method. The program also calculates the distance in pixels between the points from which the grey values where extrapolated, as well as minimum, maximum and mean grey values and the standard error of the mean. It then gives the possibility to convert the pixel distance between grey values into the real world distance by drawing a line along the scale of the image used. All the grey values, distances and calculation results are exported into an csv file named after the image used. A jpg image such as Fig. 2b is also exported as a reference.

### Rationale of working with greyscale values of BSE images

The high-resolution back-scattered electron (BSE) image of the clinopyroxene reported in Fig. 2a,b has been chosen as an example to demonstrate that the greyscale values can be used, under certain circumstances (that is, negligible Ca variation, see Supplementary Fig. 1 and ref. 3), as a proxy of Mg# (Mg/(Mg+Fe) at.) values. We have performed electron microprobe spot analyses every 2–3 μm coupled with the greyscale diffusion profile along the clinopyroxene boundary layer (Fig. 2, Supplementary Figs 2–6). For each spot analysis we have calculated the number of cations per formula unit, the formula unit weight (g mol^−1^), the weight of each cation (g mol^−1^), the backscatter coefficient (ETA, refs 52, 53), and the contribution of each element to the ETA value (Supplementary Table 1). The greyscale value (that is, the brightness) of the BSE image depends upon the ETA values of the mean atomic number of the sample volume interacting with the electron beam. Thus, the formula unit weight of each spot analysis strictly correlates with the corresponding grey value. In Supplementary Figs 1–6, we have reported the ETA value of each spot analysis versus the ETA fraction of the most relevant cations making up the formula unit of clinopyroxene, along with their relative linear regressions. In the example reported in Supplementary Fig. 1, the clinopyroxene ETA value ranges from 0.1446 to 0.1489 and the ETA fraction of Si+Al (at. wt. 28.09, 26.98 g mol^−1^) has some 3% variation from core to rim (0.0415–0.0427, Supplementary Table 1). Ca (at. wt. 40.08 g mol^−1^) has an ETA fraction variation from core (0.0370) to rim (0.0336) <10% and is negatively correlated with the ETA value of each spot analysis, meaning that Ca cannot cause the increase of brightness of the BSE image (Supplementary Fig. 1a). The ETA fraction of Fe+Mn+Ni (at. wt. 55.85, 54.94, 58.69 g mol^−1^) has a strong positive correlation with the ETA value (slope=2.55, Supplementary Fig. 1a) with >100% variation from core to rim (0.0096–0.0204, Supplementary Table 1), providing evidence for its major contribution to brightness variation of BSE image. The ETA fraction of Mg (at. wt. 24.31 g mol^−1^) is, as Ca, negatively correlated with the ETA value of each spot analysis (Supplementary Fig. 1a), and has a variation from core (0.0119) to rim (0.0139) of some 15% (Supplementary Table 1). Consequently, Mg# has a robust (*r*^2^=0.9994) and strong negative (slope=−32.9) correlation with the ETA values (Supplementary Fig. 1b). Overall, these observations set constraints on Fe (Mn, Ni)—Mg exchange as the primary cause of brightness variation of BSE image.

We have further explored the causes of brightness variation of clinopyroxene BSE image calculating the fitting profile along with the fitting parameter 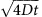 (see ‘Curve fitting protocol and timescale calculation’ for details) to the diffusion profile of Mg# (Fig. 2c), grey values (Fig. 2d), ETA values (Supplementary Fig. 1c), and Ca cations per formula unit (Supplementary Fig. 1d).

As expected, the fitting profile results of Mg#, grey values and ETA values are identical within error (Fig. 2c,d and Supplementary Fig. 1c), although the best fitting profile is that of grey values because of the extremely fine (<0.1 μm) spatial resolution (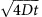
=2.63±0.17, 2 s.d., Fig. 2d). This means that if the timescale (*t*) were known, the calculated diffusion coefficients *D* would be the same. Since the only meaningful diffusion coefficient is that related to Fe–Mg exchange (that is Mg#, Fig. 2c), it is straightforward to conclude that both the grey values and the ETA values (Fig. 2d and Supplementary Fig. 1c) can be used as a proxy of Fe–Mg exchange in clinopyroxene.

In contrast, the fitting profile of Ca (Supplementary Fig. 1d) is still locked at the initial boundary conditions because this element, as much as Al, has a diffusion coefficient in clinopyroxene slower than Fe–Mg exchange^26^. This result sets constraints that the compositional zoning profile of clinopyroxenes in Fig. 1 is caused by Fe–Mg diffusion across the boundary layer and not by growth or mixing processes. Admittedly, the fitting profile of Ca is poor (ca. 70% error on 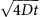) because of the intrinsic difficulty of having a reliable result with no data close to the flex point of the error function. The extremely fine spatial resolution (<0.1 μm) is, however, one of the advantages of our NIDIS model, permitting a significant improvement on the error of the fitting parameter 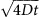 (Fig. 2d), in particular when the length of the diffusion profile is only a few microns (for example, some 5 μm, Fig. 2).

### *T* estimations

Temperature estimates of Stromboli volcano has been accurately determined on the basis of melt inclusions and experimental petrology studies^28^,^29^,^30^,^31^. The LP feeding magma has *T*=1150±10 °C whilst the HP resident magma has *T*=1098±15 °C. The clinopyroxene-liquid geothermometer of Putirka^34^ was used to check the clinopyroxene-liquid equilibrium and the consistency with temperature estimates from literature. The equilibrium test (*K*_D_(Fe–Mg)^cpx−liq^=0.27±0.03, ref. 34) indicates that the low-Mg# core and rim are in equilibrium with the HP magma^27^, whereas the high-Mg# band is in equilibrium with the LP magma^27^. The calculated temperatures of the low-Mg# cores and rims have been 1030–1060 °C, whereas those of the high-Mg# bands have been 1115–1150 °C. For the purpose of this paper, focused on reporting the rationale of the NIDIS model, we worked with temperatures of 1098±15 °C and 1150±10 °C for the low- and high-Mg# (0.72–0.76; 0.83–0.88) clinopyroxene portions, respectively^28^,^29^,^30^,^31^.

### Mineral composition

High-resolution core-rim compositional profile, ∼2–3 μm step size, of each clinopyroxene were obtained using a Cameca SX 100 electron microprobe to check the relationship between Mg# and grey values (Figs 2, 3, 4, Supplementary Figs 1–6). The Cameca SX 100 at IAC-NHM (Imaging and Analysis Center at The Natural History Museum, London) is equipped with five WDS (wavelength dispersive) spectrometers and one EDS (energy dispersive) spectrometer. It was operated at 20 KeV and 20 nA with a focused beam. Sodium was measured as first elements and counted for 10 s, all the other elements (Si, Mg, Al, Ca, Ti, Cr, Mn, Fe, Ca and Ni) were measured for 20 s. Matrix effects were corrected using the XPHI Cameca built-in protocol.

### Curve fitting protocol and timescale calculation

The grey values obtained are fitted with the Matlab script createfit, which uses the Curve Fitting Toolbox of Matlab. In particular, the Matlab function *fit()* from the toolbox is used. It allows fitting of custom functions to any data as well as the use of several fitting options. The nonlinear least squares fitting option is used for the purpose of this work. The diffusion equation across a semi-infinite plane sheet, modified after Crank^37^ (equation 2.14), has been used to fit the grey values profile of clinopyroxenes 1–4 (Fig. 4, CPX1–4) obtained as described in ‘Greyscale calculation’:


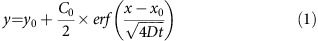


The diffusion equation across a finite reservoir of limited extent, modified after Crank^37^ (equation 2.15), has been used to fit the grey values profile of clinopyroxene 5 (Fig. 4, CPX5):


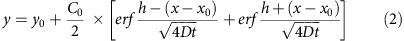


where *y* is the grey value at point *x* (μm); in equation 1, *x*_0_ and *y*_0_ are the coordinates to shift the origin of the graph to the flex point of the profile; in equation 2, *x*_0_ and *y*_0_ are the midpoint and the grey value of the finite reservoir, respectively; *C*_0_/2 is half the difference between the maximum and minimum grey value along the profile; *erf* is the error function (the script automatically decides whether to use the error function or the complementary error function fitting routine); *D* (m^2^ s^−1^) is the diffusion coefficient; *t*(s) is the time calculated by the fitting procedure; *h* (μm) is half width of the finite reservoir. In the case of equation 2, the value *C*_0_/2 has been obtained assuming the grey value of the initial boundary conditions as that of the final rim grey value (Fig. 4, CPX5, see also main text: ‘NIDIS and the lifetime history of Stromboli clinopyroxene’).

The diffusion coefficient is calculated using the Arrhenius equation:


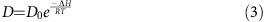


considering Fe–Mg exchange in clinopyroxene. The values of the pre-exponential factor *D*_0_ (m^2^ s^−1^) and the activation energy Δ*H* (kJ mol^−1^) are from Dimanov & Sautter^35^, *R* is the gas constant=8.3145 (J mol^−1^ K^−1^), *T* is the temperature (K).

The Matlab *fit()* function returns the best fitting values for each parameter with the uncertainty at 95% confidence level (2 s.d.).

For the purpose of this work, 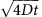 is the parameter of interest. Given the diffusion coefficient *D* from (3), the value of 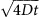 which best fits the data allows us to calculate the residence time *t* of the crystal from which the grey values are extracted. The error calculated on the parameter 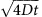 by the *fit()* function, together with the error on the temperature which affects the diffusion coefficient *D*, are then used by the createfit script to calculate the relative error propagation on the residence time *t*:


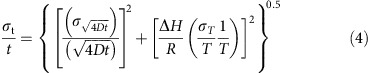


assuming that the errors of the fitting parameter 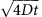 and *T* are normally distributed, independent from each other and there is negligible or no covariance between them.

The createfit script requires as input a csv file with the grey values, the distance between grey values and the errors associated with the grey values (that is, the standard error of the mean). It fits the values with either equations 1 or 2, 12 taking into account the associated errors weighing each point by:


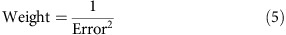


It then asks the operator to enter *D*_0_, Δ*H*, *T* and the error on *T* and, using the value for the fitting parameter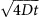 calculated by the *fit()* function and its fitting error, it calculates the residence time *t* and its absolute error (2 s.d.). In the case of equation 2, it also requires the initial grey value of the finite reservoir to be entered. Together with a reference diagram, (grey values versus distance) showing the data and the fitted curve, the script returns as Matlab variables the values associated with the fitting parameters, the residence time and its error.

### Data availability

The data that support the findings of this study are available from the corresponding author on request. The complete code, both grey values and createfit Matlab scripts along with the instructions on how to use it, can be accessed at the following address: https://github.com/cpetrone/NIDIS. All the scripts have been tested on machines running Matlab 2011b, 2014b and 2015a and Windows XP, Windows 7, Windows 10, Ubuntu 12.04 and OS 10.7, 10.10 and 10.11. Given the on going development of the code, we kindly ask users to contact the corresponding author with comments and suggestions for improvement.

## Additional information

**How to cite this article**: Petrone, C. M. *et al*. Pre-eruptive magmatic processes re-timed using a non-isothermal approach to magma chamber dynamics. *Nat. Commun.* **7**, 12946 doi: 10.1038/ncomms12946 (2016).

## Supplementary Material

Supplementary Information Supplementary Figures 1-6, Supplementary Table 1 and Supplementary References

## Figures and Tables

**Table 1:** Diffusion chronometry results of the NIDIS model.

**sample**	**CPX1 site 29**		****	**CPX2 site 7**		****	**CPX3 17 (profile1)**			**CPX4**	****		**CPX5 Finite Reservoir**		
*T (°C) 1150±10*															
core-band (μm)^a^				0–45			0–60			0–100			0–66*		
erf parameters^b^				value	2 s.d.		value	2 s.d.		value	2 s.d.		value	2 s.d.	
y0				123.8	±0.1		118.9	±0.1		121.8	±0.1		115.7	±0.1	
Co/2				9.9	±0.1		8.2	±0.1		15.4	±0.2		8.7	±0.1	
x0				13.4	±0.1		17.0	±0.1		47.0	±0.2		33.0	±0.2	
h (μm)													6.4	±0.2	
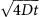 × 10^−6^				3.0	±0.1		5.2	±0.3		7.7	±0.4				
rsquares				0.9937			0.9944			0.9912			0.9467		
*T (°C) 1098±15*															
band-rim (μm)^a^	0–80			30–72			35–80			70–135			70–130		
erf parameters^b^	value	2 s.d.		value	2 s.d.		value	2 s.d.		value	2 s.d.		value	2 s.d.	
y0	139.9	±0.1		123.0	±0.1		117.5	±0.2		124.2	±0.2		123.4	±0.2	
Co/2	10.0	±0.1		8.5	±0.1		5.8	±0.2		17.1	±0.2		9.9	±0.2	
x0	34.5	±0.2		56.9	±0.1		71.5	±0.2		113.7	±0.2		100.3	±0.2	
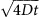 ** × **10^−6^	7.0	±0.4		2.5	±0.1		4.2	±0.4		6.1	±0.3		6.3	±0.4	
rsquares	0.9936			0.9962			0.9783			0.9912			0.9842		
															
	value	2 s.d.	2 s.d.*	value	2 s.d.	2s.d.*	value	2 s.d.	2 s.d.*	value	2 s.d.	2 s.d.*	value	2 s.d.	2 s.d.*
Δ*t*1 (yrs)				0.2	±0.2	±0.1	0.6	±0.6	±0.3	1.4	±1.2	±0.5	2.3	±1.4	±0.5
Δ*t*2 (yrs)	11.9	±4.8	±1.2	1.6	±0.6	±0.1	4.2	±1.8	±0.8	9.2	±3.7	±1.0	9.5	±3.9	±1.3
Δ*t*3 (yrs)				0.4	±0.1	±0.1	1.1	±0.4	±0.2	2.5	±0.7	±0.3	2.6	±0.7	±0.4
Δ*t*4 (yrs)				0.6	±0.2	±0.1	1.8	±0.5	±0.2	3.9	±1.0	±0.4	4.9	±1.2	±0.3
*residence time* (yrs)															
(Δ*t*1+Δ*t*2)	11.9	±4.8	±1.2	1.8	±0.7	±0.1	4.8	±1.9	±0.8	10.5	±3.9	±1.1	11.8	±4.2	±1.1

The error propagation at 95% confidence level on the time estimate is reported considering the uncertainty on both 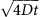 and T (2 s.d.), and only on 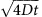 (2 s.d.*). Constants: Do=9.5 × 10^−05^ m^2^  s^−1^ and ΔH=406 kJ mol^−1^ (ref. 35); D=1098 °C=3.26 × 10^−20^±1.27 m^2^ s^−1^; D=1150 °C=1.20 × 10^−19^±0.29 m^2^ s^−1^.

^a^ Core-band and band-rim are the widths of the modelled diffusion profiles. In the case of CPX5, the core-band width is in fact the clinopyroxene portion around the light-band defining the finite reservoir of limited extent (Figs 1 and 4 CPX5).

^b^ The erf parameters are from the fitting of the diffusion profiles (equations 1 and 2).

^*^This profile has been modelled using the finite reservoir equation (equation 2) and the grey values of the initial conditions of the light band has been assumed as that of the final rim (*y*=133).
