# Supplementary material for: Pre-eruptive magmatic processes re-timed using a non-isothermal approach to magma chamber dynamics
Source: Nat Commun. 2016 Oct 5;7:12946. doi: 10.1038/ncomms12946 (PMC5476789; doi:10.1038/ncomms12946)
Supplement: Supplementary Information — Supplementary Figures 1-6, Supplementary Table 1 and Supplementary References [file ncomms12946-s1.pdf]

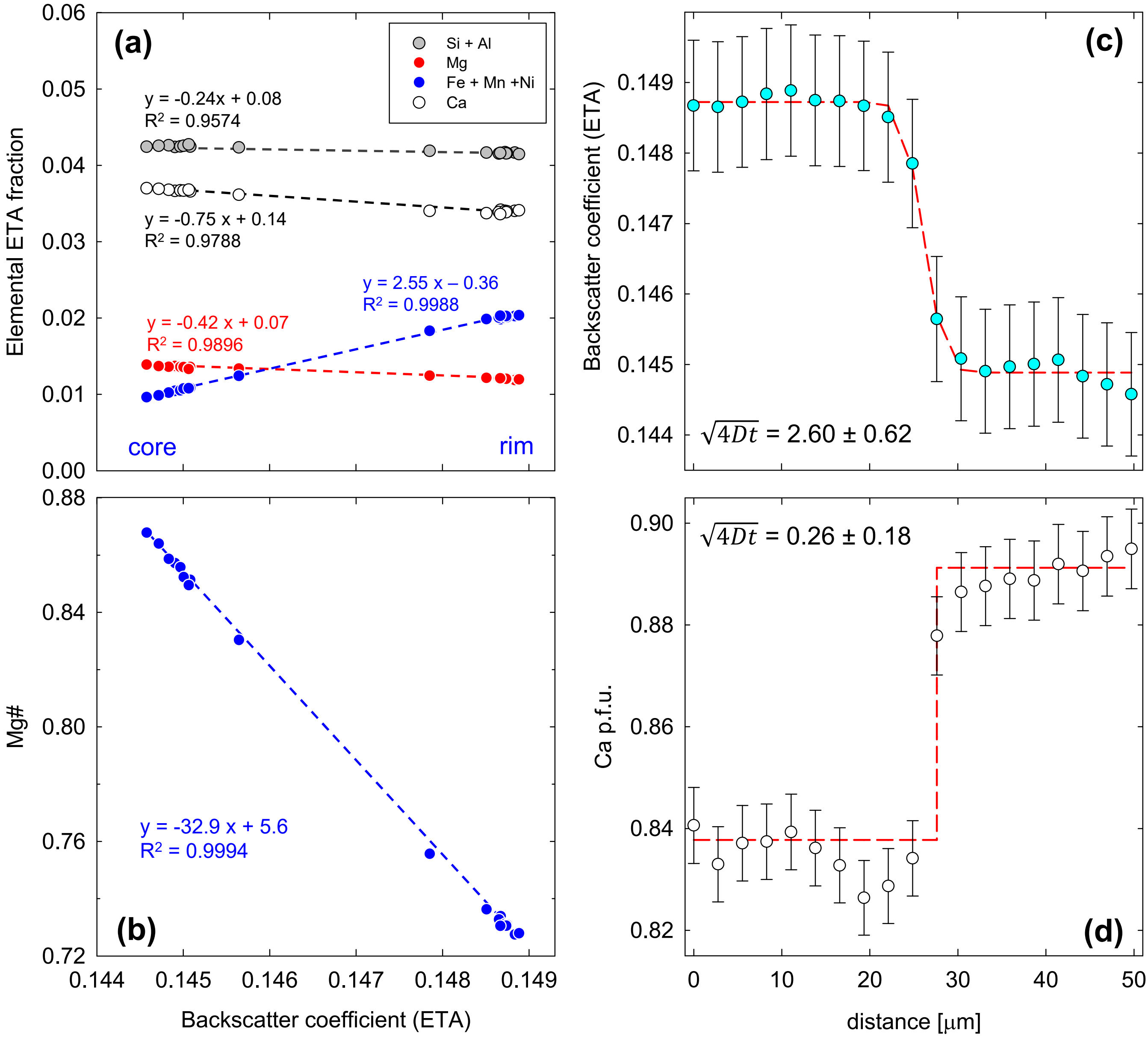

Supplementary Figure 1 – Fitted profile of clinopyroxene compositional boundary. The composition of a clinopyroxene has been measured along the red dots profile in Fig. 2a to evaluate the variation and contribution of each element to the backscatter coefficient[1,2] (ETA), i.e. to the brightness of BSE image. **a.** ETA value of each spot analysis versus ETA fraction of Si+Al, Ca, Fe+Mn+Ni, and Mg along with their relative linear regressions. **b.** ETA value of each spot analysis versus Mg# and the relative linear regression. **c.** ETA value versus distance [μm] compositional profile, along the red dots traverse of the clinopyroxene shown in Fig. 2a. The red line is the best fitting of the compositional profile and the value of the fitting parameter $\sqrt{4Dt}$ is given. **d.** Compositional profile of Ca cations (a.p.f.u.) versus distance [μm], along the red dots traverse of the clinopyroxene shown in Fig. 2a. The red line is the best fitting of the compositional profile and the value of the fitting parameter $\sqrt{4Dt}$ is given. Error bars (2 standard deviation) represent uncertainties of electron microprobe analyses (Ca), and error propagation uncertainties of electron microprobe analyses (ETA).

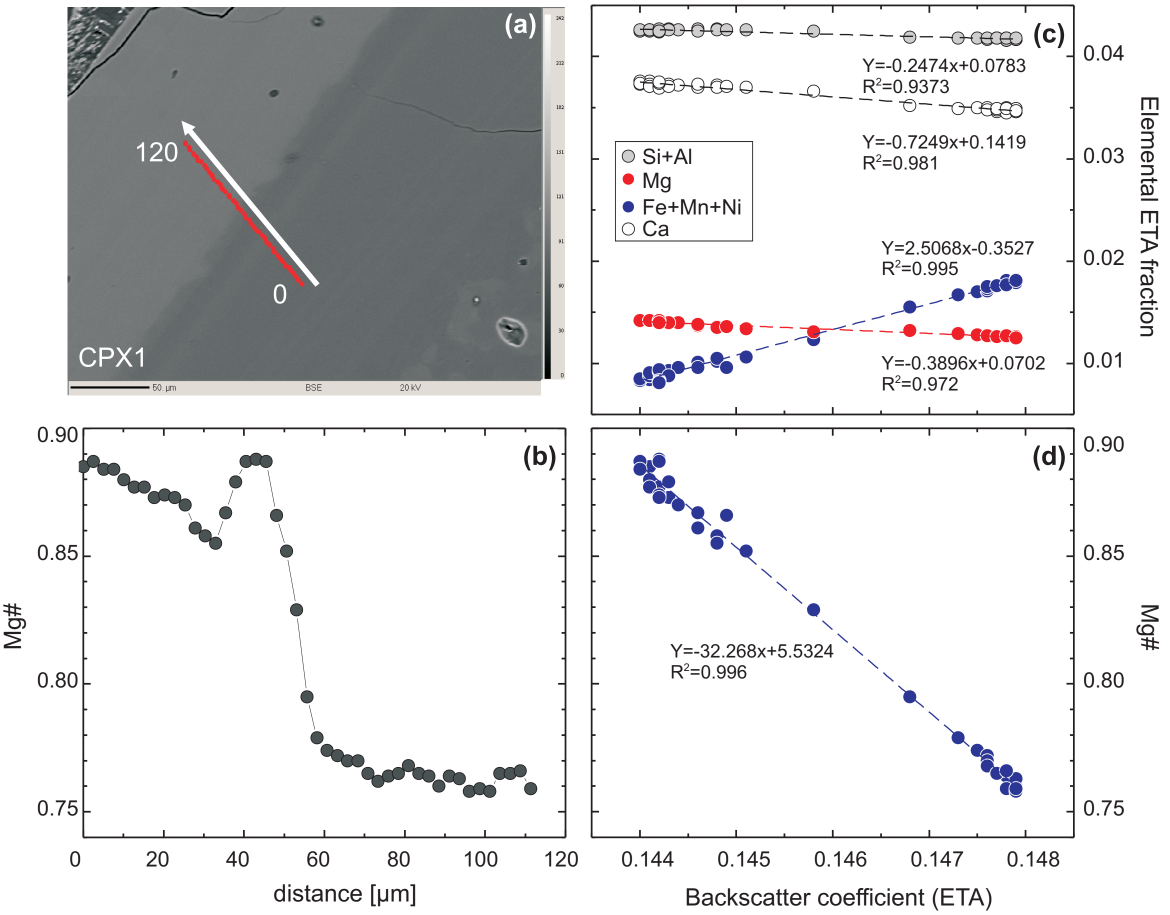

Supplementary Figure 2 - Electron microprobe compositional data of CPX1.
a. BSE image of the compositional boundary. The composition has been measured along the red dots profile. The white arrow indicate the profile direction from core to rim; **b.** Mg# versus distance [µm] along the analysed compositional boundary**. c.** ETA value of each spot analysis versus ETA fraction of Si+Al, Ca, Fe+Mn+Ni, and Mg along with their relative linear regressions. **d.** ETA value of each spot analysis versus Mg# and the relative linear regression.

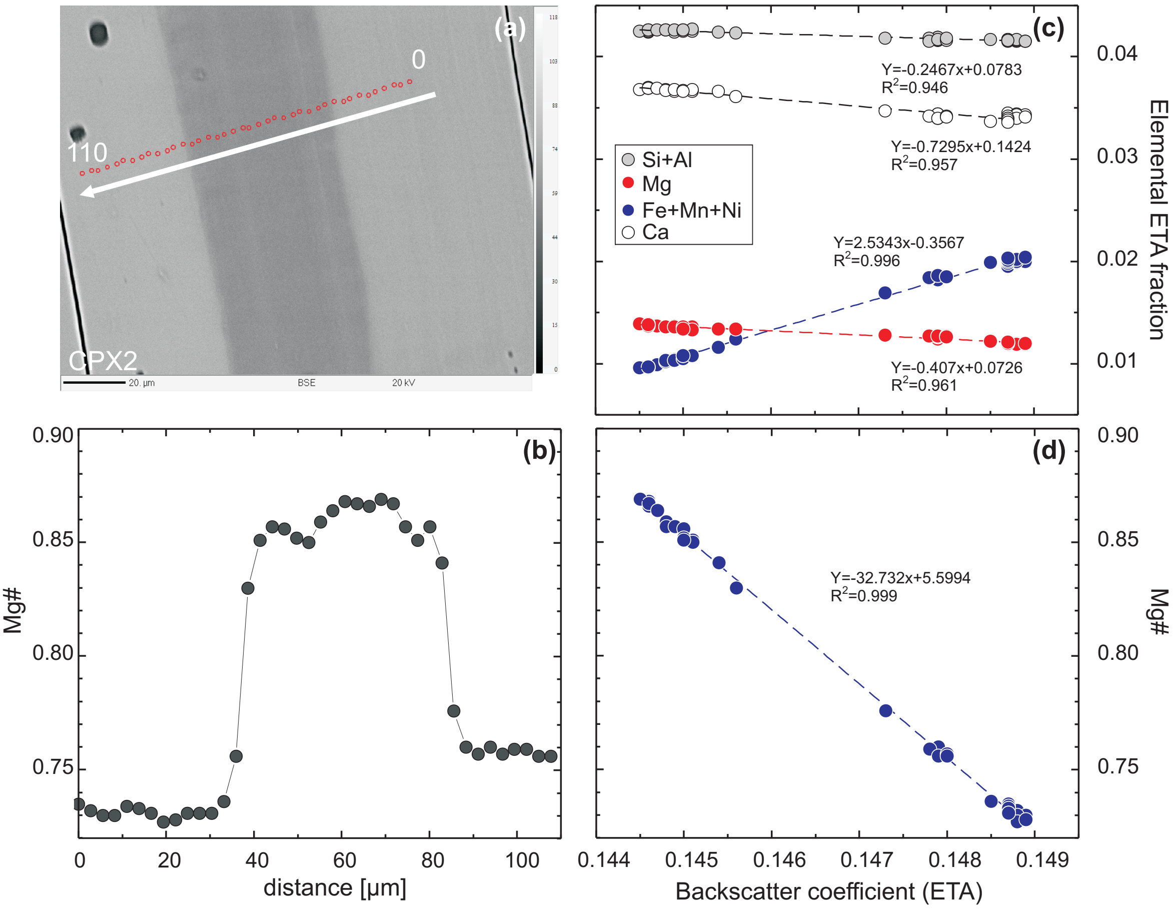

Supplementary Figure 3 - Electron microprobe compositional data of CPX2.
a. BSE image of the compositional boundary. The composition has been measured along the red dots profile. The white arrow indicate the profile direction from core to rim; **b.** Mg# versus distance [µm] along the analysed compositional boundary**. c.** ETA value of each spot analysis versus ETA fraction of Si+Al, Ca, Fe+Mn+Ni, and Mg along with their relative linear regressions. **d.** ETA value of each spot analysis versus Mg# and the relative linear regression.

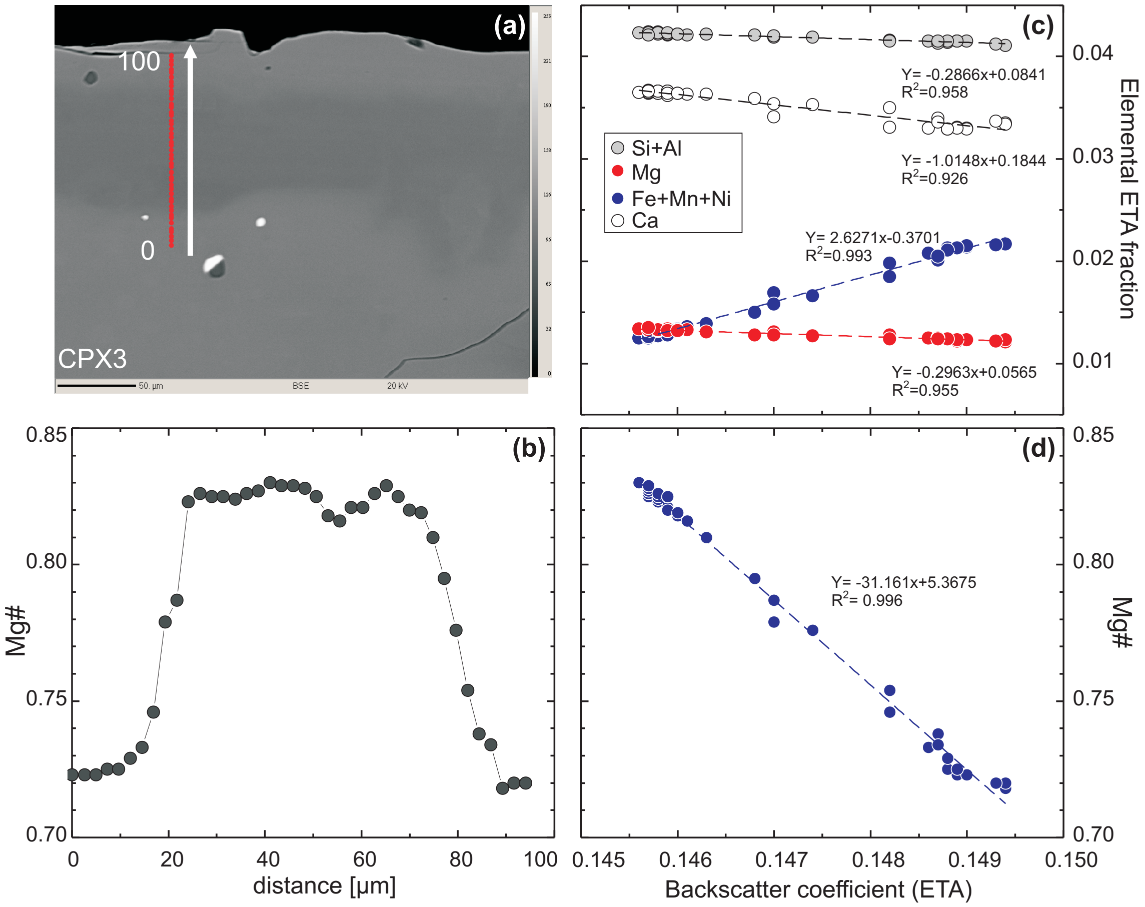

Supplementary Figure 4 - Electron microprobe compositional data of CPX3.
a. BSE image of the compositional boundary. The composition has been measured along the red dots profile. The white arrow indicate the profile direction from core to rim; **b.** Mg# versus distance [µm] along the analysed compositional boundary**. c.** ETA value of each spot analysis versus ETA fraction of Si+Al, Ca, Fe+Mn+Ni, and Mg along with their relative linear regressions. **d.** ETA value of each spot analysis versus Mg# and the relative linear regression.

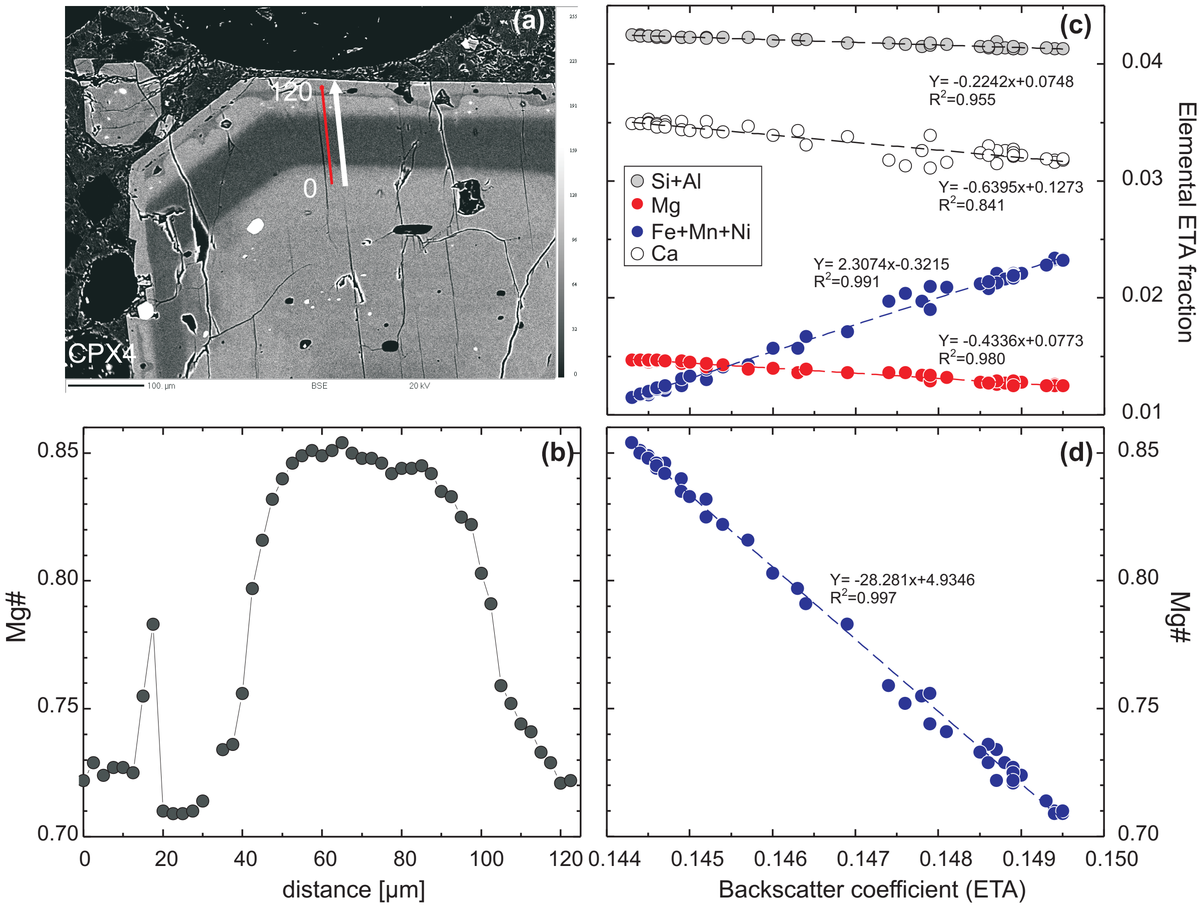

Supplementary Figure 5 - Electron microprobe compositional data of CPX4.
a. BSE image of the compositional boundary. The composition has been measured along the red dots profile. The white arrow indicate the profile direction from core to rim; **b.** Mg# versus distance [µm] along the analysed compositional boundary**. c.** ETA value of each spot analysis versus ETA fraction of Si+Al, Ca, Fe+Mn+Ni, and Mg along with their relative linear regressions. **d.** ETA value of each spot analysis versus Mg# and the relative linear regression.

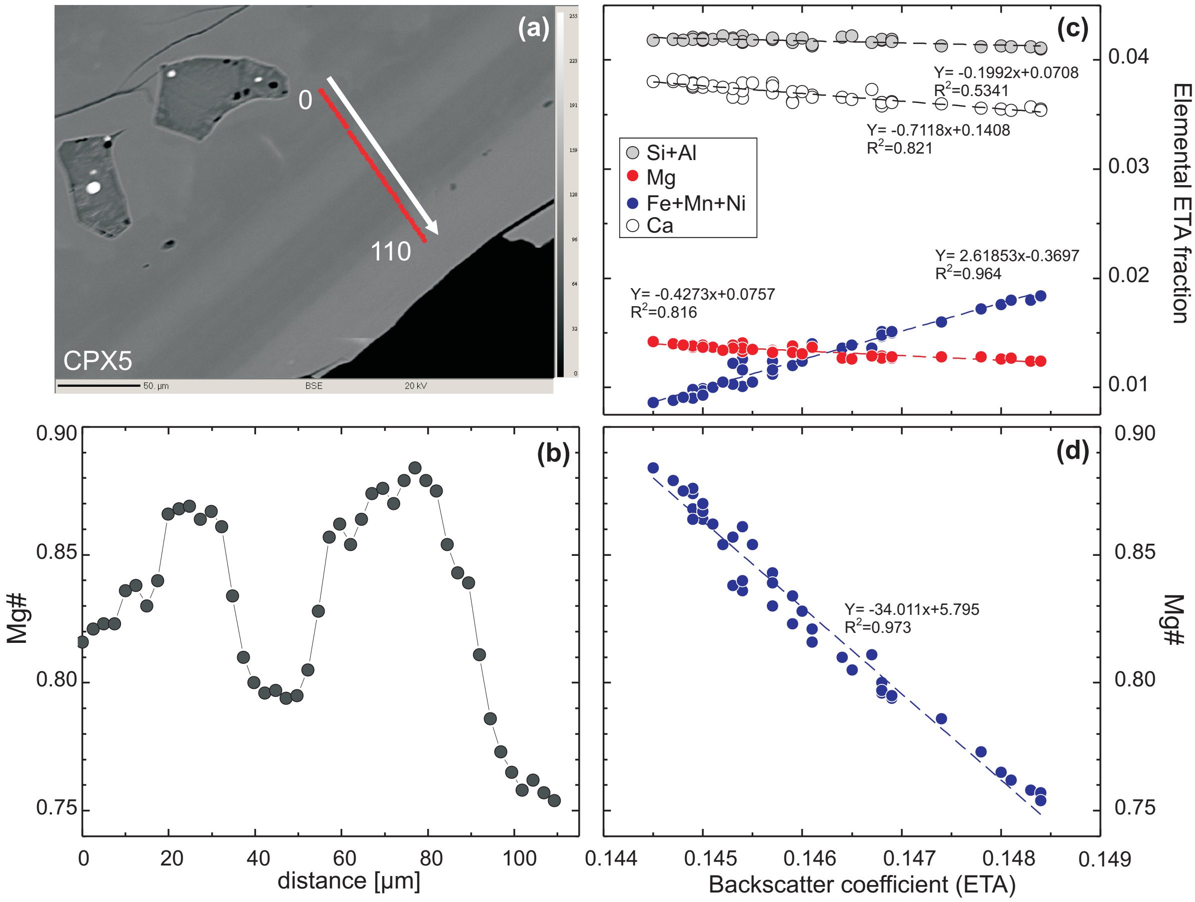

Supplementary Figure 6 - Electron microprobe compositional data of CPX5.
a. BSE image of the compositional boundary. The composition has been measured along the red dots profile. The white arrow indicate the profile direction from core to rim; **b.** Mg# versus distance [µm] along the analysed compositional boundary**. c.** ETA value of each spot analysis versus ETA fraction of Si+Al, Ca, Fe+Mn+Ni, and Mg along with their relative linear regressions. **d.** ETA value of each spot analysis versus Mg# and the relative linear regression.

Supplementary Table 1. Electron microprobe analyses of the compositional boundary layer of the clinopyroxene in Fig. 2

Major Elements

Distance (µm)	SiO_2	TiO_2	Al_2O_3	Cr_2O_3	Fe_2O_3	FeO	MnO	NiO	MgO	CaO	Na_2O	sum
0 **(rim)**	49.8	0.98	4.14	0.06	2.96	6.23	0.22	0.02	14.1	21.1	0.38	99.9
2.76	49.9	0.97	4.05	0.06	3.31	6.05	0.23	0.01	14.2	20.9	0.44	100.2
5.52	50.0	1.00	4.12	0.04	2.53	6.72	0.22	0.01	14.1	21.0	0.37	100.1
8.28	49.8	1.04	4.20	0.02	2.84	6.51	0.23	0.03	13.9	21.0	0.43	100.0
11.05	49.4	1.02	4.21	0.02	3.51	5.93	0.24	0.02	14.0	21.0	0.41	99.8
13.81	49.6	1.02	4.25	0.04	3.14	6.22	0.21	0.01	14.1	20.9	0.39	99.9
16.57	49.6	1.00	4.26	0.04	3.42	6.00	0.22	0.04	14.1	20.9	0.43	100.0
19.33	49.6	1.00	4.27	0.04	3.19	6.24	0.24	0.00	14.2	20.7	0.41	99.9
22.09	49.8	1.00	4.21	0.05	3.08	6.12	0.22	0.04	14.3	20.8	0.43	100.0
24.85	50.2	1.04	4.28	0.05	2.56	5.91	0.23	0.02	14.7	21.1	0.35	100.4
27.62	51.6	0.48	3.54	0.45	1.77	4.02	0.12	0.01	15.8	22.4	0.26	100.3
30.38	51.6	0.45	3.63	0.59	1.66	3.39	0.10	0.02	16.0	22.6	0.24	100.3
33.14	51.6	0.44	3.64	0.62	2.07	2.85	0.10	0.03	16.2	22.7	0.25	100.6
35.9	51.6	0.45	3.71	0.60	1.84	3.03	0.13	0.05	16.0	22.7	0.26	100.5
38.66	51.6	0.43	3.78	0.51	1.58	3.42	0.10	0.03	16.0	22.7	0.21	100.4
41.42	51.0	0.54	4.40	0.41	1.57	3.43	0.09	0.04	15.6	22.7	0.20	100.0
44.19	51.4	0.48	4.01	0.55	1.59	3.17	0.10	0.03	16.0	22.7	0.19	100.2
46.95	51.8	0.46	3.73	0.59	1.56	3.05	0.09	0.03	16.2	22.9	0.22	100.6
49.71 **(core)**	51.5	0.42	3.71	0.59	2.07	2.49	0.10	0.01	16.4	22.9	0.20	100.3

Atomic proportion on the basis of 4 cations and Fe^{3+} from stoichiometry

Distance (µm)	Si	^{IV}Al	^{VI}Al	Ti	Cr	Fe^{3+}	Mg	Ni	Fe^{2+}	Mn	Ca	Na	Mg#	2sd
0 **(rim)**	1.853	0.147	0.035	0.028	0.002	0.083	0.783	0.000	0.194	0.007	0.841	0.028	0.734	± 0.024
2.76	1.853	0.147	0.030	0.027	0.002	0.093	0.789	0.000	0.188	0.007	0.833	0.031	0.733	± 0.024
5.52	1.859	0.141	0.040	0.028	0.001	0.071	0.780	0.000	0.209	0.007	0.837	0.026	0.731	± 0.024
8.28	1.854	0.146	0.038	0.029	0.000	0.080	0.773	0.001	0.203	0.007	0.837	0.031	0.727	± 0.024
11.05	1.844	0.156	0.029	0.029	0.001	0.099	0.780	0.001	0.185	0.008	0.839	0.029	0.728	± 0.024
13.81	1.848	0.152	0.034	0.029	0.001	0.088	0.783	0.000	0.194	0.007	0.836	0.028	0.731	± 0.024
16.57	1.846	0.154	0.033	0.028	0.001	0.096	0.784	0.001	0.187	0.007	0.833	0.031	0.731	± 0.024
19.33	1.848	0.152	0.035	0.028	0.001	0.089	0.789	0.000	0.194	0.007	0.826	0.029	0.731	± 0.024
22.09	1.851	0.149	0.036	0.028	0.002	0.086	0.791	0.001	0.190	0.007	0.829	0.031	0.736	± 0.024
24.85	1.854	0.146	0.041	0.029	0.001	0.071	0.808	0.001	0.183	0.007	0.834	0.025	0.756	± 0.025
27.62	1.889	0.111	0.041	0.013	0.013	0.049	0.860	0.000	0.123	0.004	0.878	0.018	0.830	± 0.032
30.38	1.886	0.114	0.043	0.012	0.017	0.046	0.872	0.001	0.104	0.003	0.886	0.017	0.851	± 0.034
33.14	1.881	0.119	0.038	0.012	0.018	0.057	0.880	0.001	0.087	0.003	0.888	0.018	0.857	± 0.035
35.9	1.883	0.117	0.043	0.012	0.017	0.050	0.872	0.001	0.093	0.004	0.889	0.018	0.856	± 0.035
38.66	1.885	0.115	0.048	0.012	0.015	0.043	0.871	0.001	0.104	0.003	0.889	0.015	0.852	± 0.034
41.42	1.869	0.131	0.060	0.015	0.012	0.043	0.855	0.001	0.105	0.003	0.892	0.014	0.850	± 0.034
44.19	1.877	0.123	0.050	0.013	0.016	0.044	0.872	0.001	0.097	0.003	0.891	0.014	0.859	± 0.035
46.95	1.885	0.115	0.045	0.012	0.017	0.043	0.878	0.001	0.093	0.003	0.893	0.015	0.864	± 0.036
49.71 **(core)**	1.879	0.121	0.038	0.011	0.017	0.057	0.890	0.000	0.076	0.003	0.895	0.014	0.868	± 0.036

Backscatter coefficient (ETA) and elemental ETA fraction per formula unit

Distance (µm)	Si	Al	Ti	Cr	Fe	Mg	Ni	Mn	Ca	Na	Ox	ETA	2sd
ETA 15keV	0.1644	0.1529	0.2454	0.2629	0.2794	0.1412	0.2949	0.2713	0.2268	0.1291	0.0911		
0 **(rim)**	0.0383	0.0034	0.0014	0.0001	0.0193	0.0120	0.0000	0.0005	0.0342	0.0004	0.0391	0.1487	± 0.0009
2.76	0.0383	0.0033	0.0014	0.0001	0.0196	0.0121	0.0000	0.0005	0.0339	0.0004	0.0391	0.1487	± 0.0009
5.52	0.0384	0.0033	0.0015	0.0001	0.0195	0.0120	0.0000	0.0005	0.0340	0.0004	0.0391	0.1487	± 0.0009
8.28	0.0383	0.0034	0.0015	0.0000	0.0197	0.0119	0.0001	0.0005	0.0340	0.0004	0.0391	0.1488	± 0.0009
11.05	0.0380	0.0034	0.0015	0.0000	0.0198	0.0120	0.0001	0.0005	0.0341	0.0004	0.0391	0.1489	± 0.0009
13.81	0.0381	0.0034	0.0015	0.0001	0.0196	0.0120	0.0000	0.0004	0.0340	0.0004	0.0391	0.1487	± 0.0009
16.57	0.0381	0.0034	0.0015	0.0001	0.0197	0.0120	0.0001	0.0005	0.0338	0.0004	0.0391	0.1487	± 0.0009
19.33	0.0382	0.0035	0.0015	0.0001	0.0198	0.0121	0.0000	0.0005	0.0336	0.0004	0.0391	0.1487	± 0.0009
22.09	0.0383	0.0034	0.0015	0.0001	0.0193	0.0122	0.0001	0.0005	0.0337	0.0004	0.0391	0.1485	± 0.0009
24.85	0.0384	0.0035	0.0015	0.0001	0.0178	0.0124	0.0001	0.0005	0.0340	0.0003	0.0392	0.1479	± 0.0009
27.62	0.0395	0.0029	0.0007	0.0008	0.0122	0.0134	0.0000	0.0003	0.0361	0.0002	0.0396	0.1456	± 0.0009
30.38	0.0395	0.0029	0.0007	0.0011	0.0106	0.0136	0.0001	0.0002	0.0366	0.0002	0.0397	0.1451	± 0.0009
33.14	0.0395	0.0029	0.0006	0.0011	0.0102	0.0137	0.0001	0.0002	0.0366	0.0002	0.0397	0.1449	± 0.0009
35.9	0.0395	0.0030	0.0007	0.0011	0.0101	0.0136	0.0001	0.0003	0.0367	0.0002	0.0397	0.1450	± 0.0009
38.66	0.0395	0.0030	0.0006	0.0009	0.0105	0.0136	0.0001	0.0002	0.0367	0.0002	0.0397	0.1450	± 0.0009
41.42	0.0392	0.0036	0.0008	0.0007	0.0105	0.0133	0.0001	0.0002	0.0368	0.0002	0.0397	0.1451	± 0.0009
44.19	0.0394	0.0032	0.0007	0.0010	0.0099	0.0136	0.0001	0.0002	0.0368	0.0002	0.0397	0.1448	± 0.0009
46.95	0.0396	0.0030	0.0007	0.0010	0.0096	0.0137	0.0001	0.0002	0.0369	0.0002	0.0397	0.1447	± 0.0009
49.71 **(core)**	0.0394	0.0030	0.0006	0.0011	0.0094	0.0139	0.0000	0.0002	0.0370	0.0002	0.0398	0.1446	± 0.0009

Core-rim ETA fraction variation of the major constituents grouped according to their similar atomic weights

	Si+Al	Fe+Mn+Ni	Mg	Ca	Ox
absolute	0.00127	-0.0107	0.00203	0.0034	0.00066
percentage	3.0%	-111%	14.6%	9.2%	1.7%

footnote: Major element composition of the clinopyroxene in Fig. 2a, measured along the red dots profile (0 µm: rim, 50 µm: core). For each spot analysis we have calculated the number of cations per formula unit, the backscatter coefficient (ETA, Ref. 1, 2), and the contribution of each element to the ETA value. The backscatter coefficient has been calculated from the formula unit weight [g mol^{-1}], the weight of each element [g mol^{-1}], and the elemental backscatter coefficient at 15 keV (Ref. 2). The core-rim ETA fraction variation (absolute and percentage) of the major constituents of the clinopyroxene composition is also reported. These major constituents are grouped according to their similar atomic weights (Si + Al, Ca, Mg, Fe + Ni + Mn, Ox). Mg# [Mg/(Mg+Fe) at.] and ETA uncertainties (2 standard deviation) represent error propagation of electron microprobe analyses.

Supplementary References

1. Reed, S.J.B. Electron microprobe analysis. *Cambridge Univ. Press*, pp.399 (1975).
2. Hunger, H.J. & Küchler L. Measurements of the electron backscattering coefficient for quantitative EPMA in the energy range 4 to 40 keV. *IPSSa*, **56**, K45-K48 (1979).
